# Movement plans for posture selection do not transfer across hands

**DOI:** 10.3389/fpsyg.2015.01358

**Published:** 2015-09-11

**Authors:** Christoph Schütz, Thomas Schack

**Affiliations:** ^1^Faculty of Psychology and Sports Science, Bielefeld UniversityBielefeld, Germany; ^2^Cognitive Interaction Technology, Center of Excellence, Bielefeld UniversityBielefeld, Germany; ^3^CoR-Lab, Research Institute for Cognition and Robotics, Bielefeld UniversityBielefeld, Germany

**Keywords:** motor hysteresis, motor planning, posture selection, transfer, motor control

## Abstract

In a sequential task, the grasp postures people select depend on their movement history. This motor hysteresis effect results from the reuse of former movement plans and reduces the cognitive cost of movement planning. Movement plans for hand trajectories not only transfer across successive trials, but also across hands. We therefore asked whether such a transfer would also be found in movement plans for hand postures. To this end, we designed a sequential, continuous posture selection task. Participants had to open a column of drawers with cylindrical knobs in ascending and descending sequences. A hand switch was required in each sequence. Hand pro/supination was analyzed directly before and after the hand switch. Results showed that hysteresis effects were present directly before, but absent directly after the hand switch. This indicates that, in the current study, movement plans for hand postures only transfer across trials, but not across hands.

## Introduction

More than two decades ago, Rosenbaum and Jorgensen (1992) published their influential paper on the planning of macroscopic aspects of manual control. The authors demonstrated that the grasp postures people select in a sequential task depend on the previously used postures. Participants had to pick a horizontal bar from a cradle on a tabletop, place its left or right end against a target location on the front of a bookshelf, and return the bar to the cradle. This procedure was repeated for a column of 14 target locations, once in ascending and once in descending order. If participants had to place the left end on the target, they used an overhand grip for the upper and an underhand grip for the lower targets. Thus, they avoided awkward arm postures at the end of the movement. This behavior was termed the *end-state comfort effect* (Rosenbaum and Jorgensen, 1992). It has since been reproduced in a large number of studies (cf. Rosenbaum et al., 2012).

More importantly, the point-of-change between the over- and the underhand grip shifted depending on the sequence: in the descending sequences, it was located at a lower target than in the ascending sequences. Participants persisted on using the former grasp type for a number of the central drawers. The tendency of the motor system to switch from one movement state to another at different values depending on its movement history was termed *motor hysteresis* (Kelso et al., 1994). Motor hysteresis in posture selection has been replicated in several studies. For example, Weigelt et al. (2009) asked participants to open a column of slotted drawers in either ascending or descending sequence and found a similar shift of the point-of-change. By replacing the slots with cylindrical knobs, Schütz et al. (2011) extended research on motor hysteresis from a binary to a continuous posture selection task. While participants continuously adapted their posture between drawers, posture in the descending sequences of trials remained more pronated than in the ascending ones.

Motor hysteresis effects have not only been demonstrated for posture selection, but also for the selection of individual limb segments (Meulenbroek et al., 1993) and whole limbs (Weiss and Wark, 2009). A number of studies focused on hysteresis effects on the end-effector trajectory. Jax and Rosenbaum (2007, 2009) had participants execute a center-out pointing task with an obstacle present in random trials. Hand path curvature was higher if an obstacle was present in the previous trial and decayed quickly as the time between trials increased. After a 1000 ms delay between successive trials, the effect was almost completely eliminated. Diedrichsen et al. (2010) asked participants to move a horizontal line into a horizontally elongated target box. The hand position on the line was irrelevant to the task. If the hand was guided passively along this task-redundant dimension, a lasting deviation of the hand path could be induced. This deviation persisted even after the passive guidance was removed.

While motor hysteresis in some cases may derive from dynamic muscle properties, a number of studies suggest that hysteresis effects result from cognitive aspects of movement planning. In their *plan-modification hypothesis*, Rosenbaum et al. (2007) state that the planning and execution of a reaching movement is associated with a cognitive cost. This cognitive cost can be reduced by the reuse and modification of former movement plans. This reuse should draw upon working memory resources. In the drawer study by Weigelt et al. (2009), participants had to memorize a sequence of letters while conducting a sequential motor task. Under these dual-task conditions, one of the most reliable effects in memory research, the recency effect, was lost, indicating that motor performance affects working memory. Spiegel et al. (2012) further demonstrated that the re-planning of an intended movement interfered with working memory performance, and that spatial memory was affected stronger than verbal memory (Spiegel et al., 2013).

Experimental evidence for the cognitive nature of the hysteresis effect on posture selection was provided by Schütz and Schack (2013). In their *cost-optimization hypothesis*, the authors state that the motor system does not seek to minimize the cognitive cost of movement planning, but the combined cognitive and mechanical cost of movement planning and movement execution. To test this hypothesis, participants had to open a column of drawers with cylindrical knobs in a sequential order, which resulted in a hysteresis effect. If the mechanical cost for opening a drawer was increased by adding a counterforce of 25 N for 10 sequences, the size of the hysteresis effect was significantly reduced. Effect size remained diminished even after the additional mechanical cost was removed, which supports the notion that motor hysteresis results from cognitive aspects of motor planning.

Even better evidence for the cognitive nature of the hysteresis effect was provided for end-effector trajectories. Van der Wel et al. (2007) asked participants to contact a series of target disks on a tabletop in time with a metronome, either in clockwise or counter-clockwise progression. On some trials, an obstacle had to be cleared between two targets. Jump peak height after the obstacle decreased only gradually, following an exponential decay back to the original height. More importantly, this hysteresis effect persisted even if participants cleared the obstacle with one hand and continued the progression with the other, thus discarding dynamic muscle properties as the sole explanation for motor hysteresis.

To date, a transfer of the hysteresis effect across hands has only been demonstrated for the end-effector trajectory. We therefore asked whether a similar transfer would be present for the hysteresis effect on posture selection. To this end, we replicated the sequential, continuous posture selection task of Schütz and Schack (2013), which has been shown to reliably elicit hysteresis effects on posture selection. The original task was restricted to movement execution with the right hand. In the first step, we had to test whether grasp postures would differ between hands. To this end, participants had to open and close a column of drawers in randomized sequences of trials with their left and right hand, respectively.

Schütz et al. (2011) showed that hand pro/supination in a similar task was adjusted continuously with drawer (height) and did not differ between the dominant and non-dominant hand. We therefore expected similar results in the current study, that is, a significant main effect of the factor “drawer” and no main effect of the factor “hand”. Furthermore, hand pro/supination in the study of Schütz et al. (2011) did not depend on the previously grasped drawer, indicating that randomized sequences of trials did not induce hysteresis effects. Therefore, the two tasks in the current study could be executed in a fixed order, as the execution of the randomized task would not affect the hysteresis effect in the subsequent, ordered task.

In the second step, we asked whether hysteresis effects would transfer across hands. Participants were asked to open and close the drawers in ascending and descending sequences of trials. In each sequence, participants had to perform a side-step and continue the sequence with the contra lateral hand. Hand switches were inserted either before drawer #3, #4, or #5. At these drawers, the hysteresis effect was most pronounced in previous experiments (Schütz et al., 2011; Schütz and Schack, 2013).

Based on the findings by van der Wel et al. (2007), we hypothesized that hysteresis effects would transfer across hands and, thus, still be present after a hand switch. This would result in a significant main effect of the factor “order” (ascending/descending) on the hand pro/supination directly after the hand switch. We further expected a main effect of the factor “drawer”, similar to previous experiments (Schütz et al., 2011; Schütz and Schack, 2013). For drawer #4, the three different hand switch positions resulted in sequences where the hand switch occurred directly before and directly after the drawer. Therefore, if hysteresis effects were not transferred or only partially transferred, we expected a significant interaction of the factors “order” and “time of hand switch” (before/after drawer #4).

## Materials and methods

### Design

The experiment consisted of two tasks. In task 1, participants executed randomized sequences of trials (cf. Table 1a). The manipulated factors were “hand” (left/right) and “drawer” (#3/#4/#5). The factor “hand” did not affect the grasp posture and, thus, was eliminated from task 2 by averaging.

**Table 1:** **Sequences used in the (1) randomized and (2) ordered task**.

**Task**	**Order**	**Hand switch**	**Sequence**											**Repetitions (per hand)**
1	Randomized		(a)	e.g.,	6	3	7	8	5	1	2	4	9	3
		3	(b)	1	2	^*^	3	4	5	6	7	8	9	2
	Ascending	4	(c)	1	2	3	^*^	4	5	6	7	8	9	2
		5	(d)	1	2	3	4	^*^	5	6	7	8	9	2
2		3	(e)	9	8	7	6	5	4	^*^	3	2	1	2
	Descending	4	(f)	9	8	7	6	5	^*^	4	3	2	1	2
		5	(g)	9	8	7	6	^*^	5	4	3	2	1	2

* *denotes a hand switch*.

In task 2, participants executed ordered (ascending/descending) sequences of trials. A hand switch was part of each sequence, either before drawer #3, #4, or #5 (see Table 1b–g). Two analyses were conducted on the data set of task 2:

First, the difference in grasp posture depending on movement direction was analyzed directly after the hand switch. The manipulated factors were “order” (ascending/descending) and “drawer” (#3/#4/#5). A factor “hand switch position” was not included, as it was identical to the factor “drawer”.

Second, the difference in grasp posture depending on movement direction was analyzed at drawer #4 directly before and directly after the hand switch. To this end, two new factors were created from the existing sequences (see Table 1b–g), “order” and “time of hand switch”. The levels of the factor “order” were “ascending” (Table 1c,d) and “descending” (Table 1e,f). The levels of the factor “time of hand switch” were “before drawer #4” (Table 1c,f) and “after drawer #4” (Table 1d,e).

### Participants

Twenty-four students (13 female and 11 male, age 25.0 ± 2.4 years) from Bielefeld University participated in the experiment in exchange for course credit. Twenty-two participants were right handed (handedness score 0.95 ± 0.12) and two were mixed handed (handedness score 0.01 ± 0.22) according to the revised Edinburgh inventory (Oldfield, 1971). Participants reported no known neuromuscular disorders and were naïve to the purpose of the study. Before the experiment, each participant provided written informed consent and read a detailed set of instructions on the task. The study was approved by the local ethics committee and in accordance with the ethical standards laid down in the sixth revision (Seoul, 2008) of the 1964 Declaration of Helsinki.

### Apparatus

**Figure 1:**
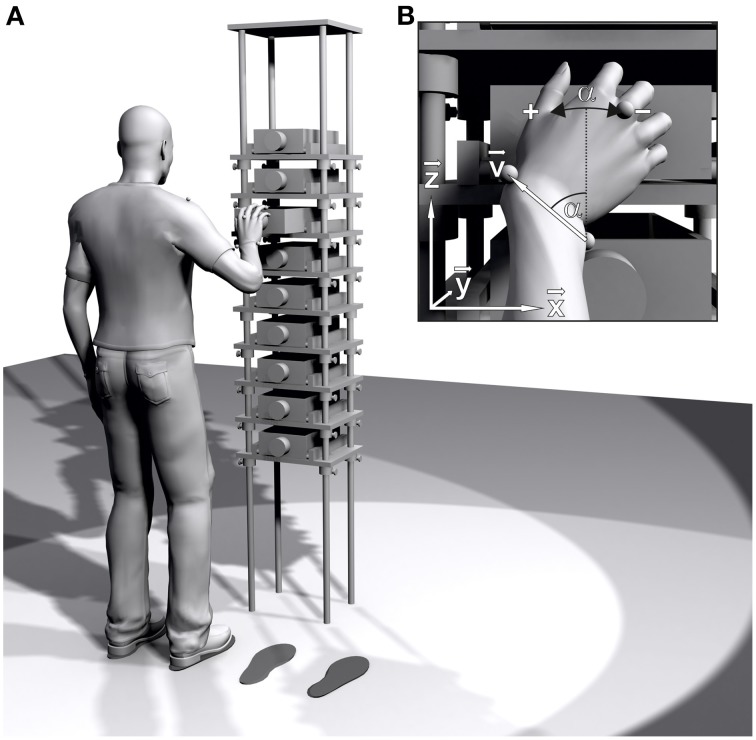
**(A)** Schematic of the experimental setup. Drawer height, drawer spacing, and participant's positions are scaled based on shoulder height and arm length. Black footprints mark the participant's positions for left/right handed task execution. **(B)** Pro/supination angle α at the moment of drawer grasp. The projection of the wrist vector **v** onto the drawer face (**x**-**z**-plane) is used to calculate α.

The apparatus used was a tall metal frame (222 cm high, 40 cm wide, and 30 cm deep) with nine wooden shelves (see Figure 1A). A wooden drawer (8.5 cm high, 20 cm wide, and 30 cm deep) was placed on each shelf, with a number from 1 (lowest) to 9 (highest) inscribed on the left side. A stop mechanism allowed for a maximum pullout range of 21.5 cm. To the center of each drawer front, a cylindrical plastic knob with a diameter of 7 cm and a depth of 4 cm was affixed.

### Preparation

Each participant was tested individually. All reflective materials (e.g., watches, jewelry) had to be removed before the experiment. Retro reflective markers (diameter 14 mm) were attached to eight bony landmarks of the thorax and both hands via palpation (see Table 2).

**Table 2:** **Anatomical landmarks used for the kinematic model**.

**Abbreviation**	**Description**
LAC	*Left articulatio acromioclaviculare*
LRS	*Left processus styloideus radii*
LUS	*Left processus styloideus ulnae*
LMC	*Left os metacarpale tertium* (dorsal of the *capitulum*)
RAC	*Right articulatio acromioclaviculare*
RRS	*Right processus styloideus radii*
RUS	*Right processus styloideus ulnae*
RMC	*Right os metacarpale tertium* (dorsal of the *capitulum*)

The participant was positioned in front of the apparatus, with the arms stretched horizontally to the side and the palms pointing toward the setup. The average height of the shoulder joint centers (0.97 × *height of L/RAC*, see Table 2) and the average arm length (distance between L/RAC and L/RRS, see Table 2) were measured to normalize the setup to the body size of the participant. The center of drawer #7 was aligned to the average height of the shoulder joint centers. Drawer spacing was set to 0.25 × *average arm length*. The participant was positioned with the shoulder joint center 1.00 × *average arm length* in front of the drawer face, once with the right shoulder joint center 0.33 × *average arm length* to the left of the drawer center and once with the left shoulder joint center 0.33 × *average arm length* to the right of the drawer center (see Figure 1A). Black cardboard footprints were used to mark the left and right position of the participant in front of the apparatus.

The left position directly replicated the participants' position in the study by Schütz and Schack (2013). From the left position, participants could execute the task comfortably with their right hand. From the right position, participants could do the same with their left hand. In theory, identical joint angles could be adopted in the left and right arm for the same drawer (height). Due to the offset of the left and right position in front of the apparatus, participants had to execute a side-step when asked to switch hands.

### Procedure

The experiment consisted of two tasks executed in a fixed order: (1) a randomized and (2) an ordered task. In the randomized task, participants executed six sequences of trials. Each sequence consisted of a pseudo-random permutation of the nine drawers (cf. Table 1a). In the ordered task, participants executed 24 sequences of trials. In half of the sequences, the nine drawers were presented in ascending order (see Table 1b-d). In the other half of the sequences, the nine drawers were presented in descending order (see Table 1e-g). Each sequence included a hand switch, either before drawer #3, #4, or #5 (see Table 1b-g). In both tasks, half of the sequences were started with the left or right hand, respectively.

Before the experiment, a pseudo-randomized list of sequences was created for each participant, based on the Mersenne twister algorithm (Matsumoto and Nishimura, 1998). The list had two sections, one for the randomized task, with six pseudo-random sequences of drawer numbers, and one for the ordered task, with 24 ordered (ascending/descending) sequences of drawer numbers. For the randomized task, a maximum of two identical repetitions were allowed for the factor “hand” (left/right). For the ordered task, a maximum of two identical repetitions were allowed for the factors “hand” (left/right), “order” (ascending/descending), and “drawer”/“hand switch position” (#3/#4/#5).

Before each sequence of trials, the participant was positioned on the left or right floor marks. Each trial was started from an initial position, with the arm hanging loosely on the side of the body and the palm of the hand touching the thigh. After the announcement of a drawer number, the participant had to (1) raise the arm to the drawer, (2) close the fingers around the knob, (3) open the drawer to the full extent, (4) close the drawer, and (5) return the arm to the initial position. As soon as the arm was back in the initial position, the experimenter announced the next drawer number. The procedure was repeated until all nine drawers of a sequence had been attended to. In the ordered sequences of trials, the experimenter also announced a “hand switch” after one of the drawers. The participant performed a side-step onto the contra lateral floor marks and continued the sequence with the other hand.

After a resting period of 30 s, the participant started the next sequence of trials. After the randomized block and halfway through the sequential block, an additional resting period of 2 min was introduced. On average, a single trial lasted for 3990 ± 660 ms in the randomized blocks and for 3140 ± 600 ms in the sequential blocks. The hand switch in the sequential blocks on average took an additional 1540 ± 550 ms. The entire experiment lasted for approximately 45 min.

### Kinematic analysis

Movement data were recorded using an optical motion capture system (Vicon Motion Systems, Oxford, UK) consisting of 12 MX-F20 CCD cameras with 200 Hz temporal and approximately 0.25 mm spatial resolution. The laboratory's coordinate system was defined with the **x**-axis pointing to the right, the **y**-axis pointing to the front and the **z**-axis pointing upwards while standing in front of the apparatus (see Figure 1B). Cartesian coordinates of the eight retro reflective markers were calculated from the camera data via triangulation. Marker trajectories were manually labeled in Vicon Nexus 1.8.5 and exported to MATLAB (2014a, The MathWorks, Natick, MA) for post processing.

For the calculation of the global pro/supination angle, the projection of the wrist axis onto the drawer face (**x**-**z**-plane) was used (see Figure 1B). A direction vector **v** was defined, pointing from the *ulnar styloid process* to the *radial styloid process* (**v** = L/RRS–L/RUS). The pro/supination angle α of the hand was calculated based on the vector components v_x_ and v_z_, using the four-quadrant inverse tangent function integrated into MATLAB. For the left hand, the sign of v_x_ was inverted to render the pro/supination angles comparable to those of the right hand. The pro/supination angle was zero when the back of the right/left hand pointed directly to the right/left side (and, therefore, **v** pointed directly upward). Pronation of the hand caused an increase, supination a decrease of the pro/supination angle.

### Data analysis

To identify the moment of drawer grasp for each trial, the trajectory of the **y**-component (perpendicular to the drawer face, see Figure 1B) of the *capitulum* marker (L/RMC) was analyzed. Each trajectory started from a low initial value, corresponding to the initial posture of the participant, and exhibited two local maxima before returning to the initial value. The time of the first local maximum, corresponding to the moment of drawer grasp, was used to extract the pro/supination angle α of the hand.

For each of the 24 participants, 270 pro/supination angles were measured, corresponding to 54 trials (2 hands × 9 drawers × 3 repetitions) in the randomized task and 216 trials (2 [starting] hands × 2 orders × 3 hand switch positions × 9 drawers × 2 repetitions) in the sequential task.

For the statistical analysis, repeated measures analyses of variance (rmANOVAs) were conducted on the pro/supination angles. All factors were within-subjects variables. Repetition was not included as a factor. Instead, the repetitions of each factor combination were averaged to reduce variance. Where appropriate, the Greenhouse-Geisser correction was applied to the *p*-values. Degrees of freedom are reported uncorrected.

## Results

### Randomized task

To test for differences in posture between the left and right hand, the grasp postures used in the randomized task at drawers #3, #4, and #5 were analyzed. A 2 (hand: left/right) × 3 (drawer: #3/#4/#5) rmANOVA was conducted on the pro/supination angles.

The main effect of “drawer” was significant, *F*_(2, 46)_ = 175.456, *p* < 0.001, η^2^ = 0.603. A linear contrast calculated on the factor “drawer” was significant, *F*_(1, 46)_ = 350.885, *p* < 0.001, η^2^ = 0.603, and explained 99.99 % of the sum of squares (Type III) of the factor in the rmANOVA. Participants used a pronated grasp posture at drawer #5 (34.06°), a more neutral posture at drawer #4 (4.93°), and a supinated posture at drawer #3 (−23.23°). Neither the main effect of “hand”, *F*_(1, 23)_ = 1.522, *p* = 0.230, η^2^ = 0.003, nor the interaction of “hand” × “drawer” was significant. Participants' selected postures did not differ between the left (3.55°) and right (6.99°) hand. Therefore, the factor “hand” was averaged for the subsequent analyses.

### Ordered task

Before the experiment we hypothesized that hysteresis effects on posture selection would transfer across hands, as has previously been found for hysteresis effects on hand trajectories. To analyze whether hysteresis effects were transferred across hands, the grasp postures used at drawers #3, #4, and #5 in the ascending and descending sequences of trials were compared directly after the hand switch. A 2 (order: ascending/descending) × 3 (drawer: #3/#4/#5) rmANOVA was conducted on the pro/supination angles. The factor “hand” was averaged to reduce variance.

**Figure 2:**
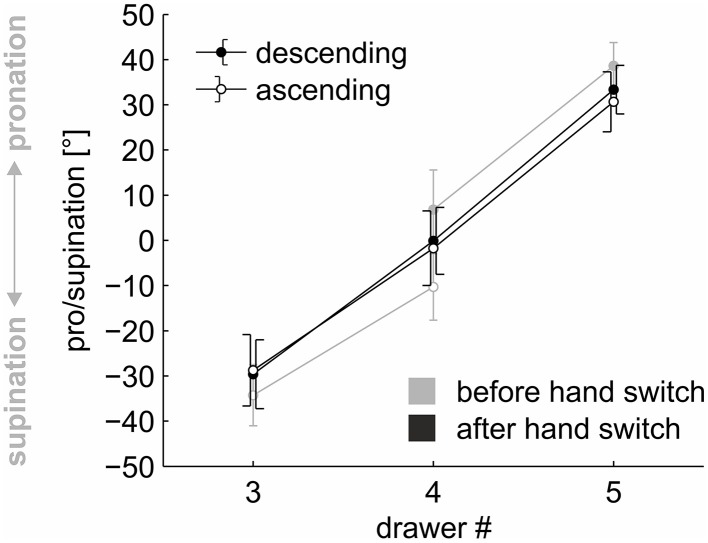
**Pro/supination angle for the ascending and descending sequences of trials at drawers #3, #4, and #5**. *Black lines* depict postures after a hand switch, *gray lines* depict postures before a hand switch. *Each data point* represents the average across the factors “hand”, “repetition”, and “participant”. *Error bars* indicate 95 % confidence intervals.

Again, the main effect of “drawer” was significant, *F*_(2, 46)_ = 254.465, *p* < 0.001, η^2^ = 0.687. A linear contrast calculated on the factor “drawer” was significant, *F*_(1, 46)_ = 507.936, *p* < 0.001, η^2^ = 0.686, and explained 99.80 % of the sum of squares (Type III) of the factor in the rmANOVA. Participants used a pronated grasp posture at drawer #5 (α = 32.01°), a more neutral posture at drawer #4 (−0.92°), and a supinated grasp posture at drawer #3 (α = −29.17°, see Figure 2, black graph). Neither the main effect of “order”, *F*_(1, 23)_ = 0.487, *p* = 0.492, η^2^ < 0.001, nor the interaction of “order” × “drawer” was significant. Participants' selected postures did not differ between the ascending (0.06°) and descending (1.21°) sequences of trials. Thus, the hysteresis effect was absent after a hand switch.

This absence of a hysteresis effect after the hand switch might have resulted from a general absence of hysteresis in the selected task. We therefore compared the grasp postures used at drawer #4 in the ascending and descending sequences if the hand switch occurred directly before or after the drawer. A 2 (order: ascending/descending) × 2 (time of hand switch: before/after drawer #4) rmANOVA was conducted on the pro/supination angles. The factor “hand” was averaged to reduce variance.

**Figure 3:**
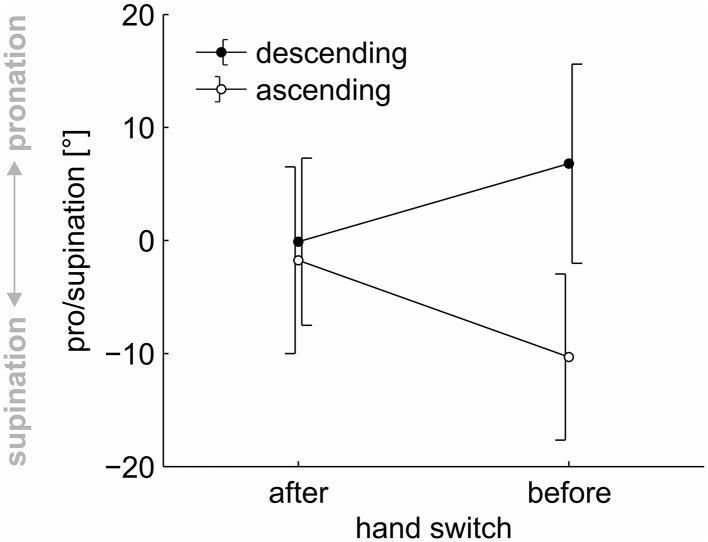
**Pro/supination angle for the ascending and descending sequences of trials at drawer #4, before and after a hand switch**. *Each data point* represents the average across the factors “hand”, “repetition”, and “participant”. *Error bars* indicate 95 % confidence intervals.

The main effect of “time of hand switch” was not significant, *F*_(1, 23)_ = 0.189, *p* = 0.668, η^2^ < 0.001. The main effect of “order” was significant, *F*_(1, 23)_ = 17.860, *p* < 0.001, η^2^ = 0.058. Participants used a more pronated grasp posture in the descending sequences (3.34°) than in the ascending sequences (−6.03°). This effect, however, was moderated by a significant interaction of “order” × “time of hand switch”, *F*_(1, 23)_ = 26.434, *p* < 0.001, η^2^ = 0.039. Paired, two-tailed *t*-tests showed that grasp posture differed significantly depending on sequence before the hand switch, *t*_(23)_ = 5.273, *p* < 0.001, but no longer after the switch, *t*_(23)_ = 0.837, *p* = 0.411 (see Figure 3). Thus, a hysteresis effect was present before the hand switch but did not transfer to the contra lateral hand after the hand switch.

## Discussion

In the current study, we asked whether former movement plans for hand postures would not only transfer between subsequent trials within the same hand, but also across hands. To this end, we designed a sequential, continuous posture selection task. Participants had to open a column of drawers with cylindrical knobs in a sequential order and switch hands once within each sequence. Results showed that a motor hysteresis effect was present directly before the hand switch, indicating a reuse of the former movement plan within the same hand. After the hand switch, the hysteresis effect was absent. This indicates that, in the current study, movement plans for hand postures do not transfer across hands.

In the randomized task, we analyzed whether grasp postures differed between the left and right hand. Results showed no effect of the factor “hand” on the selected pro/supination angle. Differences in grasp postures between hands have previously been analyzed in a number of studies on end-state comfort sensitivity. Janssen et al. had right handed (2009) and left handed (2011) participants grasp two CD cases simultaneously and place them into a CD rack (Janssen et al., 2009, 2011). In both groups, end-state comfort sensitivity was more pronounced for the right hand. However, several subsequent experiments were unable to reproduce such differences in end-state comfort sensitivity (Weigelt et al., 2006; Hughes and Franz, 2008; Seegelke et al., 2011).

Most studies investigating grasp posture selection in a sequential task were restricted to the dominant hand (Rosenbaum and Jorgensen, 1992; Short and Cauraugh, 1997, 1999; Weigelt et al., 2009; Schütz and Schack, 2013). So far, only one study analyzed differences in posture between hands (Schütz et al., 2011). The authors asked participants to open a column of drawers with cylindrical knobs in a randomized sequence with the dominant or non-dominant hand. The selected postures did not differ depending on the hand. In the current study, comparable results were reproduced for the randomized sequences of trials. This supports the notion that, in a sequential task, grasp posture selection does not differ between the left and right hand.

The main aim of the current study was to determine whether movement plans for posture selection would transfer between hands. To this end, we analyzed the postures for the ascending and descending sequences of trials directly before and directly after a hand switch. Results showed a significant interaction of the factors “order” and “time of hand switch”, with a significant hysteresis effect before and a loss of the hysteresis effect after the hand switch. The significant hysteresis effect before the hand switch indicates that posture plans were reused between subsequent trials within the same hand (Rosenbaum et al., 2007). This result is in accordance with previous research on hysteresis effects on posture selection, which found such a reuse for both binary (Rosenbaum and Jorgensen, 1992; Weigelt et al., 2009) and continuous (Schütz et al., 2011; Schütz and Schack, 2013) posture selection.

More importantly, however, the loss of the sequential effect after the hand switch indicates that there was no transfer of movement plans for posture selection across hands. This is in contrast to previous findings of van der Wel et al. (2007). In their study, participants had to contact a series of target disks on a tabletop in a sequential progression and clear an obstacle placed between two targets. Jump peak height after the obstacle decreased only gradually, which indicated a reuse of the previous movement plan. The increase in peak height transferred across hands if participants cleared the obstacle with one hand and continued the progression with the other. So why did we not find a similar transfer of posture plans across hands in the current study?

A number of differences between both studies might be responsible for this disparity. In the study by van der Wel et al. (2007), the peak jump height was analyzed in Cartesian space, i.e., in extrinsic coordinates of the workspace. Early studies on targeted limb movements found several invariant characteristics of the hand trajectories in Cartesian space, which were not found in joint space (Hogan, 1984; Atkeson and Hollerbach, 1985). These findings suggest that movement planning takes place on a higher, extrinsic level. In the current study, however, we measured the hand posture in joint space, i.e., in intrinsic coordinates on a lower level. Thus, if reaching movements were planned in Cartesian space, a transfer of the former posture to the contra lateral hand might be impossible.

In recent years, however, there has been increasing evidence that motor planning takes place in joint space, with an emphasis on the goal posture. Harris and Wolpert (1998) found that the invariant characteristics of the hand trajectories in extrinsic coordinates also result if only the variance of the goal position is minimized under the assumption of signal-dependent noise on the joint control signals. On a neurophysiological level, Graziano et al. (2002, 2005) showed that microstimulation of the motor cortex in monkeys evoked complex final postures, regardless of the required movement direction or joint torques. This result implies that final postures are encoded in the motor cortex. Thus, posture plans should be transferable to the contra lateral arm.

If movements are planned with an emphasis on the goal postures, another difference between the current study and the study by van der Wel et al. (2007) was the time of measurement. Whereas van der Wel et al. measured the hand position between target disks, posture in the current study was measured on the target position. Diedrichsen et al. (2010) demonstrated that hysteresis effects can be easily evoked in Cartesian space if the effect is limited to a task-irrelevant direction. We would like to propose a similar model for joint space, where the amount of hysteresis depends on each joint's relevance for the task goal. If strict control of a joint is required for the satisfaction of the task goal, the fraction of novel planning is increased and the hysteresis effect reduced accordingly.

In the study by van der Wel et al. (2007), for example, the jump height between two targets could be considered irrelevant to the task. The responsible joint for the jump height, the elbow, would require almost no novel planning between trials and, thus, might be highly susceptible to hysteresis effects. Even directly on the target, loose control of the elbow joint would be compensated to a certain degree by the physical contact with the target disk. In the current task, hand orientation between drawers was irrelevant to the task goal, but the hand had to be aligned to the drawer handle during the approach phase to successfully grasp the drawer. This might have resulted in a higher fraction of novel planning than in the study by van der Wel et al., which in turn might have eliminated the hysteresis effect after a hand switch.

Alternatively, the loss of the hysteresis effect might simply have resulted from the additional 1540 ms spent between subsequent trials while executing the side-step in front of the setup. In the center-out pointing task by Jax and Rosenbaum (2007, 2009), hand path curvature not only depended on the presence or absence of an obstacle in the previous trial, but also decayed quickly over time. After a 1000 ms delay, the information was almost completely lost. This matches neuropsychological and electrophysiological evidence that visual information is processed by two separate neural streams (Goodale and Milner, 1992). The streams differ in the time span over which they retain information. Information processed by the ventral stream is retained over a 48 week time span (Cave, 1997).

In contrast, information mediated by the dorsal stream, such as the sensorimotor transformations for visually guided actions, are retained for a short amount of time only. Garofeanu et al. (2004) found that object naming could be primed over a time course of several minutes, whereas object grasping (mediated by the dorsal stream) could not. Cant et al. (2005) further showed that the priming of visually guided grasp actions with a delay of 1250 ms did not affect the movement initiation time. The experiment by Jax and Rosenbaum (2009) therefore was the first to successfully demonstrate priming of the dorsal stream, albeit priming effects deteriorated after 1000 ms.

In the current study, hysteresis effects were present before the hand switch in the sequential task, even though the average delay between successive trials was 3140 ms. This result implies that posture selection was not mediated by the dorsal stream. Instead, it might rely on stored posture representations, as suggested by the *knowledge model* (Rosenbaum et al., 1993a,b). The model calculates new postures from a set of previously stored postures by a diffusion algorithm (Jax et al., 2003). As more recent postures are favored candidates for the start of the diffusion algorithm, the model also accounts for the hysteresis effect.

The knowledge model does not state the rate of decay of the recent posture information. A good estimate for the minimum retention period was provided by Weigelt et al. (2009). The authors had participants open drawers in ascending or descending sequence. From each drawer, participants had to retrieve a cup, memorize a letter inscribed on the bottom of the cup, and put the cup back into the drawer. While the authors did not report the additional time for this secondary memory task, one can safely assume that it took at least 1500 ms. Still, a significant hysteresis effect was found for the motor task. This indicates that recent posture memory does not decay fast enough to be eliminated by the additional time needed for the hand switch in the current study.

The absence of a hysteresis effect after the hand switch can therefore most likely be attributed to the fact that posture plans do not transfer across hands. As a first step in the knowledge model (Rosenbaum et al., 1993a,b), suitable postures have to be selected for the extrinsic target coordinates. The most recent goal posture, which is encoded in intrinsic coordinates, in theory is still suitable for the contra lateral arm after the hand switch. However, the side-step conducted in parallel with the hand switch changes the extrinsic target coordinates. The mismatch between the new extrinsic and the former intrinsic coordinates might cause the motor system to discard the recent posture. To avoid this, one would have to design a sequential posture selection task that maintains the extrinsic target coordinates during the hand switch, but that can still be executed comfortably with both the left and right hand.

To sum up, in the current study, we used hysteresis effects as a tool to measure whether movement plans for posture selection would transfer between subsequent trials within the same hand and between hands. Motor hysteresis effects were present directly before a hand switch, but absent directly after a hand switch. This result indicates that, while posture plans transfer from one trial to the next within the same hand, no such transfer across hands is possible.

### Conflict of interest statement

The authors declare that the research was conducted in the absence of any commercial or financial relationships that could be construed as a potential conflict of interest.

## References

[B1] Atkeson C. G. Hollerbach J. M. (1985). Kinematic features of unrestrained vertical arm movements. J. Neurosci. 5, 2318–2330. 4031998 10.1523/JNEUROSCI.05-09-02318.1985 PMC6565321

[B2] Cant J. S. Westwood D. A. Valyear K. F. Goodale M. A. (2005). No evidence for visuomotor priming in a visually guided action task. Neuropsychologia 43, 216–226. 10.1016/j.neuropsychologia.2004.11.008 15707906

[B3] Cave C. B. (1997). Very long-lasting priming in picture naming. Psychol. Sci. 8, 322–325. 10.1111/j.1467-9280.1997.tb00446.x16885147

[B4] Diedrichsen J. White O. Newman D. Lally N. (2010). Use-dependent and error-based learning of motor behaviors. J. Neurosci. 30, 5159–5166. 10.1523/JNEUROSCI.5406-09.2010 20392938 PMC6632748

[B5] Garofeanu C. Króliczak G. Goodale M. A. Humphrey G. K. (2004). Naming and grasping common objects: a priming study. Exp. Brain Res. 159, 55–64. 10.1007/s00221-004-1932-z 15221162

[B6] Goodale M. A. Milner A. D. (1992). Separate visual pathways for perception and action. Trends Neurosci. 15, 20–25. 10.1016/0166-2236(92)90344-8 1374953

[B7] Graziano M. S. A. Taylor C. S. R. Moore T. (2002). Complex movements evoked by microstimulation of precentral cortex. Neuron 34, 841–851. 10.1016/S0896-6273(02)00698-0 12062029

[B8] Graziano M. S. A. Aflalo T. N. S. Cooke D. F. (2005). Arm movements evoked by electrical stimulation in the motor cortex of monkeys. J. Neurophysiol. 94, 4209–4223. 10.1152/jn.01303.2004 16120657

[B9] Harris C. M. Wolpert D. M. (1998). Signal-dependent noise determines motor planning. Nature 394, 780–784. 10.1038/29528 9723616

[B10] Hogan N. (1984). An organizing principle for a class of voluntary movements. J. Neurosci. 4, 2745–2754. 6502203 10.1523/JNEUROSCI.04-11-02745.1984 PMC6564718

[B11] Hughes C. M. L. Franz E. A. (2008). Goal-related planning constraints in bimanual grasping and placing of objects. Exp. Brain Res. 188, 541–550. 10.1007/s00221-008-1387-8 18443769

[B12] Janssen L. Beuting M. Meulenbroek R. Steenbergen B. (2009). Combined effects of planning and execution constraints on bimanual task performance. Exp. Brain Res. 192, 61–73. 10.1007/s00221-008-1554-y 18751972

[B13] Janssen L. Meulenbroek R. G. Steenbergen B. (2011). Behavioral evidence for left-hemisphere specialization of motor planning. Exp. Brain Res. 209, 65–72. 10.1007/s00221-010-2519-5 21184219 PMC3035772

[B14] Jax S. A. Rosenbaum D. A. (2007). Hand path priming in manual obstacle avoidance: evidence that the dorsal stream does not only control visually guided actions in real time. J. Exp. Psychol. Hum. Percept. Perform. 33, 425–441. 10.1037/0096-1523.33.2.425 17469977

[B15] Jax S. A. Rosenbaum D. A. (2009). Hand path priming in manual obstacle avoidance: rapid decay of dorsal stream information. Neuropsychologia 47, 1573–1577. 10.1016/j.neuropsychologia.2008.05.019 18597796 PMC2700342

[B16] Jax S. A. Rosenbaum D. A. Vaughan J. Meulenbroek R. G. J. (2003). Computational motor control and human factors: modeling movements in real and possible environments. Hum. Factors 45, 5–27. 10.1518/hfes.45.1.5.27226 12916579

[B17] Kelso J. A. S. Buchanan J. J. Murata T. (1994). Multifunctionality and switching in the coordination dynamics of reaching and grasping. Hum. Mov. Sci. 13, 63–94. 10.1016/0167-9457(94)90029-9

[B18] Matsumoto M. Nishimura T. (1998). Mersenne twister: a 623-dimensionally equidistributed uniform pseudo-random number generator. ACM Trans. Model. Comput. Simul. 8, 3–30. 10.1145/272991.272995

[B19] Meulenbroek R. G. J. Rosenbaum D. A. Thomassen A. J. W. M. Schomaker L. R. B. (1993). Limb-segment selection in drawing behavior. Q. J. Exp. Psychol. A 46, 273–299. 10.1080/14640749308401047 8316638

[B20] Oldfield R. C. (1971). The assessment and analysis of handedness: the Edinburgh inventory. Neuropsychologica 9, 97–113. 5146491 10.1016/0028-3932(71)90067-4

[B21] Rosenbaum D. A. Cohen R. G. Jax S. A. Weiss D. J. van der Wel R. (2007). The problem of serial order in behavior: Lashley's legacy. Hum. Mov. Sci. 26, 525–554. 10.1016/j.humov.2007.04.001 17698232

[B22] Rosenbaum D. A. Chapman K. M. Weigelt M. Weiss D. J. van der Wel R. (2012). Cognition, action, and object manipulation. Psychol. Bull. 138, 924–946. 10.1037/a0027839 22448912 PMC3389205

[B23] Rosenbaum D. A. Engelbrecht S. E. Bushe M. M. Loukopoulos L. D. (1993a). Knowledge model for selecting and producing reaching movements. J. Mot. Behav. 25, 217–227. 10.1080/00222895.1993.9942051 12581991

[B24] Rosenbaum D. A. Engelbrecht S. E. Bushe M. M. Loukopoulos L. D. (1993b). A model for reaching control. Acta Psychol. 82, 237–250. 10.1016/0001-6918(93)90014-I 8475768

[B25] Rosenbaum D. A. Jorgensen M. J. (1992). Planning macroscopic aspects of manual control. Hum. Mov. Sci. 11, 61–69. 10.1016/0167-9457(92)90050-L9210854

[B26] Schütz C. Schack T. (2013). Influence of mechanical load on sequential effects. Exp. Brain Res. 228, 445–455. 10.1007/s00221-013-3576-3 23727830

[B27] Schütz C. Weigelt M. Odekerken D. Klein-Soetebier T. Schack T. (2011). Motor control strategies in a continuous task space. Motor Control 15, 321–341. 21878687 10.1123/mcj.15.3.321

[B28] Seegelke C. Hughes C. M. L. Schack T. (2011). An investigation into manual asymmetries in grasp behavior and kinematics during an object manipulation task. Exp. Brain Res. 215, 65–75. 10.1007/s00221-011-2872-z 21938544

[B29] Short M. W. Cauraugh J. H. (1997). Planning macroscopic aspects of manual control: end-state comfort and point-of-change effects. Acta Psychol. 96, 133–147. 10.1016/S0001-6918(97)00006-1 9210854

[B30] Short M. W. Cauraugh J. H. (1999). Precision hypothesis and the end-state comfort effect. Acta Psychol. 100, 243–252. 10.1016/S0001-6918(98)00020-1 9894689

[B31] Spiegel M. A. Koester D. Schack T. (2013). The functional role of working memory in the (re-)planning and execution of grasping movements. J. Exp. Psychol. Hum. Percept. Perform. 39, 1326–1339. 10.1037/a0031398 23339349

[B32] Spiegel M. A. Koester D. Weigelt M. Schack T. (2012). The costs of changing an intended action: movement planning, but not execution, interferes with verbal working memory. Neurosci. Lett. 509, 82–86. 10.1016/j.neulet.2011.12.033 22230898

[B33] van der Wel R. P. Fleckenstein R. M. Jax S. A. Rosenbaum D. A. (2007). Hand path priming in manual obstacle avoidance: evidence for abstract spatiotemporal forms in human motor control. J. Exp. Psychol. Hum. Percept. Perform. 33, 1117–1126. 10.1037/0096-1523.33.5.1117 17924811

[B34] Weigelt M. Kunde W. Prinz W. (2006). End-state comfort in bimanual object manipulation. Exp. Psychol. 53, 143–148. 10.1027/1618-3169.53.2.143 16909939

[B35] Weigelt M. Rosenbaum D. A. Hülshorst S. Schack T. (2009). Moving and memorizing: Motor planning modulates the recency effect in serial and free recall. Acta Psychol. 132, 68–79. 10.1016/j.actpsy.2009.06.005 19591968

[B36] Weiss D. J. Wark J. (2009). Hysteresis effects in a motor task with cotton-top tamarins (*Sanguinus oedipus*). J. Exp. Psychol. Anim. Behav. Process. 35, 427–433. 10.1037/a0013964 19594287

